# Mixtures of Ether and Chloroform—The Vienna Mixture

**Published:** 1873-06

**Authors:** 


### ARTICLE IV.

### *Mixtures of Ether and Chloroform—The Vienna Mixture.*

About two years after the introduction of Chloroform, a mixture of ether and chloroform began to be employed. We do not know who first introduced this compound. We ourselves were using it in 1850; but whether independently or in the track of some other experimentalist, we cannot at this moment recall. The proportions of ether and chloroform advocated at first were equal parts by measure, and the mixture was inhaled from a sponge or from Snow's inhaler. From the outset, Snow, who during his life was properly accepted as the best experimentalist and practitioner in the department of anæsthesia, was opposed to the mixture of ether and chloroform. His opposition was based on very sensible reasons. Ether, he said, is about six times as volatile as chloroform—that is to say, if equal measure of each be placed in two evaporating dishes, kept side by side, at the same temperature, the ether evaporates in about one-sixth the time of the chloroform; and when the two liquids are mixed, although they then evaporate together, the ether is converted into vapor much more rapidly. In whatever proportions they are combined, he argued further, before the whole is evaporated, the last portion of the liquid is nearly all chloroform. The consequence of this, he continued, is that at the commencement of the inhalation the vapor inspired is chiefly ether, and towards the end nearly all is chloroform, the patient experiencing the stronger pungency of ether when it is most objectionable, and inhaling the more powerful vapor at the conclusion, when there is most need to continue cautiously.

It is worthy of observation, in parenthesis, that the above admonition given by Snow, in the same sense if not in the same words, twenty years ago, and repeated by him in the words we have quoted in 1858, has been independently re-stated by Mr. Spencer Wells in the present year. Speaking of the employment of the mixture of ether and chloroform in cases of ovarian operation, Mr. Wells says: "I tried a mixture of chloroform and ether in various proportions, but soon became aware that the patient was at fiast only affected by the lighter vapor of ether, and was then subjected to the action of the chloroform just as she was least able to bear it."

The criticism passed upon the mixture of chloroform and ether at first by Snow, though itself exceedingly correct, would possibly have carried but little weight had distinct success attended the practice, and had any definite proportion of the two fluids been decided upon by competent authority. A mixture called the Vienna mixture, containing six parts of ether to two of chloroform, was of definite strength; but the mixture in our own hands and in the hands of other administrators proved uncertain, and therefore lost ground. We administered in one case of removal of the lower jaw, in two cases of ovariotomy, in one case of amputation of the leg, and in various minor operations. Report of its use from the continent was favorable; it was used it was said, in Vienna eight thousand times without a casualty.

Notwithstanding many facts in support of the Vienna mixture or chloroform and ether, we are forced to admit that it is not a perfect anæsthetic. It has the vice Snow predicted of it—it is irregular in its action. It is slow in action; for, indeed, the narcotism immediately induced is from the ether, not from the chloroform, while the action of the chloroform becomes manifest later on, and in a manner most disagreeable both to the administrator and to the operator.

When we give chloroform simply, we induce a first stage or degree of narcotism, and then a second stage or degree, with some excitement, and it may be with convulsions, tetanic rigidity, retching or vomiting. During these stages the surgeon does nothing; but there succeeds a third stage —one of quietude—during which the operation is commenced, and if extreme muscular relaxation be not needed, is completed. Here all is clear practice—a direct course for an unimpeded operative procedure when once the anæsthesia has reached the proper degree.

When ether is administered simply, there is often resistance in the early stage of narcotism, then, sometimes, convulsive movement, with undue filling of the veins, overaction of arteries, flushing of the face and occasional vomiting. This stage is followed by a stage of comparative quietude, during which the operator performs his painless task.

We have drawn these stages in bold outline for the sake of giving a clear definition of the process of anæsthesia as induced by the two agents under consideration. In some instances the different stages are only imperfectly delineated, the patient passing from the first to the third degree of narcotism almost imperceptibly: but the rule is for a series of stages more or less marked and more or less prolonged.

In administering a mixture of chloroform and ether this order of degrees or stages is broken, broken for the precise reason stated by Snow—viz., the unequal volatilization and diffusion of the two substances. Thus the first and second stages of narcotism by the ether may be developed, and a third stage follow, during which the surgeon commences his operation; but as he proceeds the action of the chloroform may begin to be felt, and rigidity of muscle, or retching or vomiting, may complicate and delay his proceedings. We observed these difficulties so often that we gave up the use of ether and chloroform mixture, although no fatal accident, and we believe no special indication of danger, ever resulted from it in our hands.

It was stated in a previous paragraph that in Vienna the mixture of chloroform and ether had not, during a long experience, been attended with fatal results. It must not thereupon be inferred that no death has followed such administration. The *American Journal of the Medical Sciences* furnishes the record of a death in July, 1857. Dr. Crockett, of Wytheville, in Virginia, was about to operate on a boy five years old, to remove a tumor from the back. In order to induce anæsthesia, chloroform and ether were administered in combination, in the proportion of one part of chloroform to four of ether, the parts being determined by fluid measure. This actually was the Vienna mixture. One fluid drachm of the mixture was poured on a funel-shaped sponge, and the administration was continued by Dr. Crockett until anæsthesia was complete. The sponge was then given to Dr. Kincannon, the family physician, who sustained the narcotism, and carefully watched the pulse. As soon as the patient was insensible the operation was commenced, and was very quickly completed. Whilst Dr. Crockett's son was sponging the wound, and waiting to see if any more arteries (beyond those that had been tied) would spring, the little boy began to vomit and became pulseless. He ejected a small portion of the contents of the stomach. He was immediately placed in the prone position, the tongue was examined to see that it had not fallen forward, and the Marshall Hall method of artificial respiration was thoroughly carried out, but without avail. The patient died within three minutes after the commencement of the vomiting. Dr. Crockett remarks on this case that there was nothing whatever to account for the fatal action of the anæsthesia. The last artery that was tied yielded bright arterial blood, showing there was no asphyxia ; and anæsthesia was never carried so far as to produce stertor, while the circulation and respiration continued perfect up to the moment of the vomiting.

It is a curious circumstance that Snow, who has commented on this case, attributed the death to loss of blood. Dr. Crockett says the loss of blood was probably four ounces, certainly not six—a loss altogether insufficient to account for death from hemorrhage. Moreover, the symptoms were not those of death from hæmorrhage, but were definitely those that mark one form of death from chloroform. In plain truth, the case is typical, in an extreme degree, of the influence of chloroform upon a subject already under the influence of ether, and Snow's original observation as to the danger of using a mixture of two substances having different boiling points and different rates of diffusion of vapor, explains the cause of death faithfully. It is because the case stands out as a striking illustration of the nature of the special danger attending the use of ether and chloroform in mixture, that we have introduced it so prominently in this place.—*London Lancet.*

